# Interventions and strategies to improve social support for caregivers of children with chronic diseases: An umbrella review

**DOI:** 10.3389/fpsyt.2022.973012

**Published:** 2022-09-23

**Authors:** Jinrong Yang, Lin Lin, Yuqin Gao, Weiren Wang, Lulu Yuan

**Affiliations:** School and Hospital of Stomatology, China Medical University, Liaoning Provincial Key Laboratory of Oral Diseases, Shenyang, China

**Keywords:** social support, caregivers, children, chronic diseases, umbrella review

## Abstract

**Background:**

Social support is an important approach to improve the psychosocial health status and promote positive coping for caregivers of children with chronic diseases. Such an approach can reduce parenting stress, help resolve parenting difficulties through the use of various social support relationships.

**Methods:**

We performed an umbrella review methodology using the method of examination, analysis and synthesis of systematic reviews. A PRISMA flow diagram was used to show the search process. The Joanna Briggs Institute was used to appraise the quality of papers and a narrative synthesis was undertaken. Relevant English and Chinese systematic reviews were searched in Embase, PubMed, Web of science, OVID, CNKI, CBM, Wan Fang and Cochrane Library databases, until November 2021, June 2022.

**Results:**

Out of 1,905 records, we included fourteen systematic reviews for a synthesis. Evidence to promote social support for caregivers of children with chronic diseases was identified from four key aspects: (i) Intervention content; (ii) Intervention forms; (iii) Intervention time; and (iv) Sources of support.

**Conclusions:**

The findings of this review suggest that a combination of differing interventions, especially for early family, including the content of parenting training or education, attitude building and resource provision, which can implement online is recommend. More interventional studies and quantitative evidence syntheses are still needed.

**Impact:**

Adequate social support is essential to promote the psychological wellbeing of caregivers of children with chronic diseases. In the early stage of children's diseases, integrating different content and forms of interventions for caregivers' families and actively helping caregivers to identify available support resources can improve social support. The findings from this review can be used to guide caregivers of children with chronic diseases and provide evidence for healthcare professionals and social workers to carry out relevant interventions.

## Introduction

Adequate social support is a protective factor for the mental health of caregivers of children with chronic diseases. Since caregivers are the closest contact of children with chronic diseases, and the mental health of caregivers directly affects the quality of life and psychological condition of children with chronic diseases, the objective of this paper was to determine which interventions and strategies could promote the level of social support among caregivers of children with chronic diseases.

### Background

Chronic disease in children is defined as that: the affected population is between 0 and 18 years old; the diagnosis is based on valid and reliable professional criteria; the disease is currently incurable or very resistant to treatment; and the disease is active and has been present for at least 3 months and is expected to persist and/or relapse intermittently (1). Pediatric chronic diseases include diabetes, congenital deformities, asthma, cancer, kidney disease, pervasive developmental disorders, etc. Over the past decades, the prevalence of chronic conditions in children has increased (2, 3). Chronic diseases afflict more than 25% of American children (4). In China, about 10–20% of children suffer from chronic diseases (5). Children with chronic diseases are less happy and less fulfilled compared to healthy peers (6). In addition to this, chronic diseases in children can cause some delay in developmental milestones such as physical, social, and emotional growth (7). These delays not only affect the physical health and psychosocial condition of children but even bring disadvantages to family members and society (7).

Although caring for children with chronic diseases has also brought positive impacts in the form of a sense of achievement and benefit (8–10), it is particularly worth noting that due to the long course of the disease and the special stage of children's growth and development, the challenges faced by caregivers of children with chronic diseases remain central to the parenting process. As key players in parenting, caregivers of children with chronic diseases participate in the whole process of the disease. Caregivers need to be involved in different areas of childcare, including assisting with biomedical, physical, rehabilitation, psychological, and family health issues, and managing the social, financial, and emotional challenges that accompany chronic disease (11). During this process, their behaviors and mentality directly affect the mental health of children (12). Therefore, the mental health of caregivers of children with chronic diseases cannot be ignored. The impact and consequences of caring for families with chronical illness children is a global public health issue with implications for the psychological and relational health of caregivers. Research has shown that caring for children with chronic diseases translates the physical, psychological, socioeconomic, and behavioral impacts of caregivers into vulnerability, reducing the quality of life, life satisfaction, and wellbeing (13, 14). Caring for children with chronic diseases may also affect parents' work, family relationships, and friendships, and lead to personal stress (15).

Social support is usually defined as the social resources that persons perceive to be available or that are actually provided to them by non-professionals in the context of both formal support groups and informal helping relationships (16). It is mainly studied as perceived usability, satisfaction with usability competent support (17, 18) or seeking social support as a positive, problem-focused coping style (19). Social support is commonly associated with wellbeing and psychological growth. Taking adequate social support for caregivers of children with chronic diseases would be beneficial to relieve the pressure of parenting, reduce anxiety, depression and other negative emotions, and improve the quality of life and social function among caregivers of children with chronic diseases (20, 21).

Social support is a protective factor for caregivers of children with chronic diseases. Caregivers of children with chronic diseases have lower levels of social support and higher levels of loneliness than caregivers of healthy children (22, 23). Parents of children with autism in developing countries experience greater stress than parents in developed countries, partly due to the lack of social support systems (24). Researchers emphasize that the establishment of effective social support is beneficial to reduce the risk of mental health problems for parents of children (25). Therefore, how to improve the social support level of caregivers of children with chronic diseases deserves attention.

Studies have shown that the level of social support is related to perceived stress, caregiver mentality, educational attainment, employment status and the utilization of support (26, 27). Some systematic reviews have shown that connected health technologies are beneficial for providing psychosocial support for family caregivers affected by Pediatric cancer (28), and Early Family Intervention Program can increase perceived spousal emotional support for parents of children with appearance-affecting health condition (29). Parenting intervention on psychosocial adjustment can also improve social support for parents of children with type 1 diabetes mellitus (30). However, evidence is fragmented. There are few comprehensive syntheses of existing evidence and the umbrella review to improve social support for caregivers of children with chronic diseases has not been performed. Thus, this review aims to assess, analyze, and synthesize existing evidence for improving social support for caregivers of children with chronic diseases.

## Methods

We presented an umbrella review following the PRISMA guidelines (31) and steps in conducting an umbrella review by Aromataris et al. (32). This type of review is more specific and addresses a focused range of outcomes (33). We chose this type of review which inform decision-making and evidence-based practice in health care to summarize existing evidence but do not re-synthesize existing synthesized data. This review followed a written protocol, accessible in Supplementary material 1. Findings were reported using a narrative synthesis.

### Search strategy

A systematic search was carried out in the following databases: Embase, PubMed, Web of Science, OVID, CNKI, CBM, Wan Fang and Cochrane Library. We used specific terms embedded in each database to maximize sensitivity. Key terms searched were (“parents” OR “caregivers”) AND “social support” AND (“Meta-Analysis” OR “systematic review” OR “Meta”). The full list of search terms and search strategy in per database were provided in Supplementary material 2. Hand searches were conducted by screening reference lists of included articles. Papers published in English and Chinese related to the research topic until November 2021 were set as the search limits. An updated search performed in June 2022.

### Eligibility criteria

We used the PICo framework to define the study eligibility criteria. Population: caregivers (fathers and/or mothers) of children with chronic diseases under the age of 18; Intervention/Phenomena of interest: interventions or strategies aimed at social support; Context: In families of children with chronic diseases under the age of 18; Study design: systematic review, meta-analysis; Timeframe: until November 2021, June 2022; Language: English and/or Chinese. We excluded protocols, narrative reviews, scoping reviews or studies without full text.

### Assessment of methodological quality

Review papers included in the final analysis were critically evaluated by two authors independently using the Joanna Briggs Institute Critical Appraisal Checklist for Systematic Reviews and Research Synthesis (32). The Joanna Briggs Institute assessment tool consists of 11 questions (see **Table 2**). Each item is appraised as Yes, No, Unclear or Not applicable. Each “Yes” response gains one point, and all the other answers get zero point. Based on the sum of points, the quality of papers was divided into three groups: low quality (0–4), moderate quality (5–8) and high quality (9–11) (45). According to types and the quantity of original research, under the guidance of the JBI Evidence Pre-Grading System (46), evidence was also graded (see Supplementary material 4).

### Data extraction and synthesis

JY and LL took charge of data extraction on basis of the predefined criteria (e.g., authors, year, country, purpose, etc.). Then YG and WW checked the extracted content of the above one by one and improved the extracted information. LY was asked in case of disagreement until consensus was reached on all extracts. The synthesis was implemented as the preconcerted plan. Firstly, the first author sought for free codes in the articles involved line by line. Secondly, primary subthemes were raised by integrating these free codes. Thirdly, secondary themes were developed by comparing and analyzing the primary subthemes. Last, all the co-authors discussed and reached the consensus for the following results.

## Results

### Search outcomes

**Figure 1:**
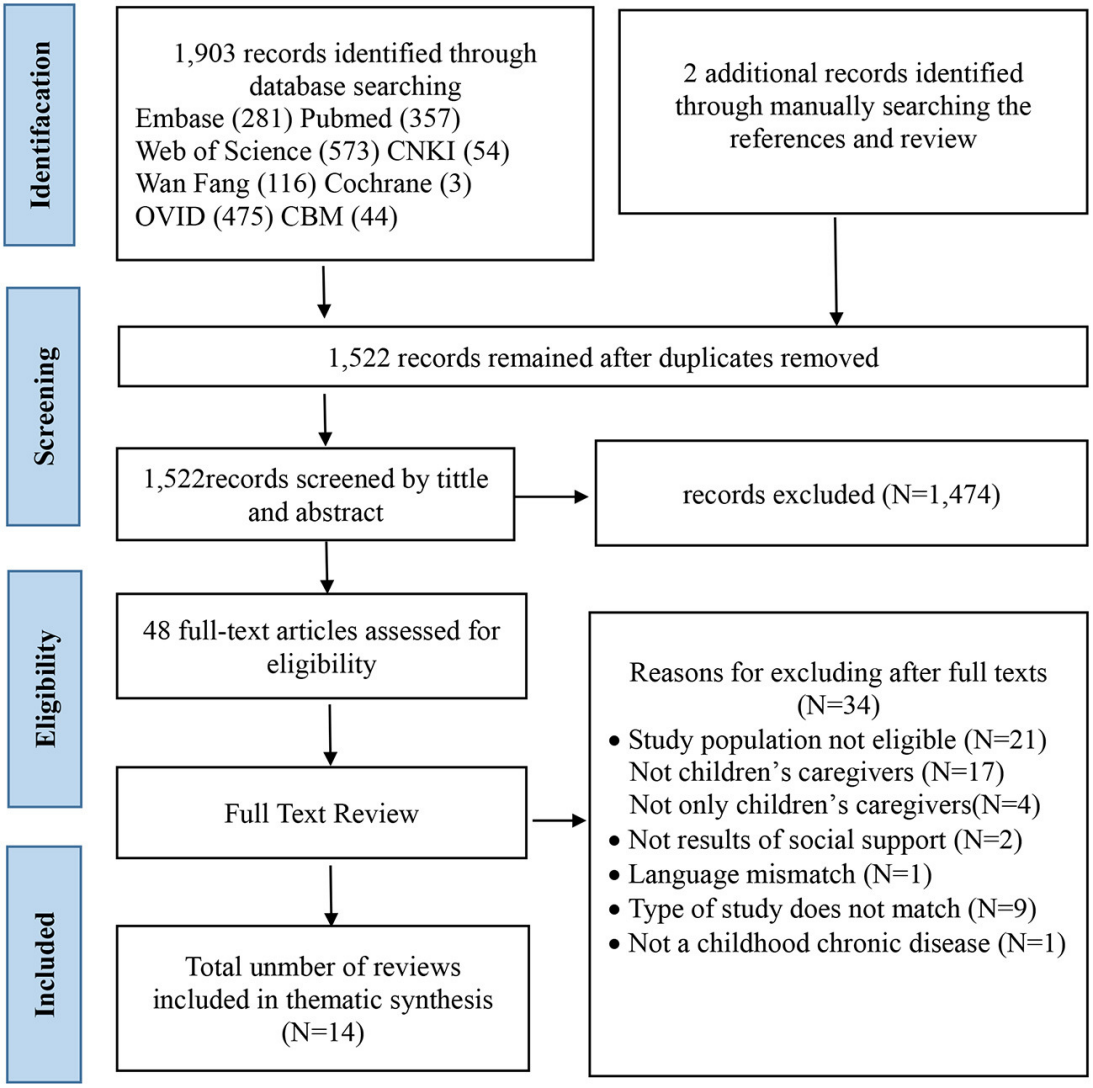
PRISMA flow diagram of included studies.

In total, 1,905 articles were identified. With the help of the reference management program EndNote X7.7, we identified and eliminated 383 duplicates. Two authors independently reviewed the titles and abstracts, those unrelated to our topic and who did not meet the inclusion criteria (*n* = 1,474) were excluded, 48 articles were included in the next stage. After the authors reviewed the full-text papers for the suitability, thirty-four non-compliant papers were excluded. Therefore, fourteen review papers were included in the final synthesis. A PRISMA flow diagram was presented in Figure 1. A list of excluded studies with reasons for exclusion can be found in Supplementary material 3.

### Characteristics of reviewed articles

Table 1 gave an overview of the included systematic reviews. Four papers were from the United Kingdom, four from the United States, two from Australia, three from China and one from Ireland. The average number of authors was 4.36 ± 2.41. The average number of databases used was 6.36 ± 3.27. The amount of articles included was 20.57 ± 11.99. The systematic reviews included children with intellectual disability, cancer, type 1 diabetes, autism, cerebral palsy, disability, congenital heart disease, and appearance-affecting health conditions. They were published between the years of 2014 and 2022.

**Table 1:** Characteristics of included reviews.

**Reference and country**	**Study design**	**Including papers (*N*)**	**PIO/PICo/PICoS**	**Search strategy**	**Theme**	**Risk of bias**	**Score**	**Quality rating**
Wilson et al. (34); United Kingdom	Systematic review	*n* = 7	P: Parents with ID I: Interventions to promote social relationships and parenting skills O: Quantitative outcome measures to judge the effectiveness of the intervention	DBs: OVID, psycINFO, EMBASE, ERIC, Medline, MIDRIS, CINAHL, ASSIA IC: In English	To review the effectiveness of interventions to strengthen social relationships and parenting skills	CQA	8/11	MQ
Gise and Cohen (35); United States	Systematic review	n=37	P: Parents of children with cancer I: Phenomenon of social support Co: Family of children with cancer	DBs: PsycINFO, CINHAL, MEDLINE IC: In English between January 2010 and May 2021	To review social support in parents of children with cancer	CQA	10/11	HQ
Costa et al. (29); United Kingdom	Systematic review	*n* = 15	P: Parents and/or guardians of children (< 18 years) with AAHC I: A psychosocial intervention C: Compare the intervention group to a control group O: Psychosocial outcomes	DBs: MEDLINE, PsychARTICLES, PsychINFO, CINAHL Plus, BND, CL IC: In English	To review the effectiveness of interventions to improve psychosocial outcomes	CQA	11/11	HQ
Kimbell et al. (36); United Kingdom	Systematic review	*n* = 14	P: Parents caring for a child aged ≤ 8 years with T1DM I: Views and/or experiences of parents caring for a young child with T1DM Co: Family of children with TIDM S: A primary research using qualitative methods or mixed-methods studies reporting qualitative data separately	DBs: Medline, EMBASE, CINAHL, PsycINFO, WoS IC: In English	To synthesize the qualitative evidence on parents experiences of caring for a child with T1DM to identify: the challenges they encounter; their views about support received; ways in which support could be improved; and directions for future research	CQA	10/11	HQ
Boehm and Carter (37); United States	Systematic review	*n* = 52	P: Parents of individuals with ID or ASD I: Relationship and/or informal relationship Co: Family of individuals with ID or ASD	DBs: ERIC, PsycINFO, SA, SSA IC: In English before July 2014	To review informal relationships of parents and their association with a range of parent and family outcomes	NQA	8/11	MQ
Nuske et al. (38); United States	Systematic review	*n* = 27	P: Students (< 18 years old) with autism spectrum disorder and their parents and teachers O: Strategies for successful student school transition	DBs: PsycINFO, ERIC IC: In English	To review the difficulties that school transitions pose for students with ASD and their parents and teachers, and the strategies used to support during school transition	CQA	10/11	HQ
Rea et al. (39); United States	Systematic review	*n* = 21	P: Parents and/or siblings of children with AAHC I: Therapeutic recreation camp O: Assessment of parent, sibling, or family outcomes	DBs: PubMed, PsycInfo, SportDISCUS, HSN/AE IC: In English between January 2000 and May 2018	To review therapeutic recreation camps impact the parents and siblings of children facing a variety of chronic health conditions.	CQA	9/11	HQ
Zhao et al. (30); China	Meta-analysis	*n* = 17	P: Parents of children or adolescents under 18 years old with T1DM I: Supportive parenting training or education programs O: Psychosocial, family-related and/or sociological outcomes	DBs: PubMed, MEDLINE, EMBASE, CINAHL, CL, WoS IC: In English from January 1978 to October 2018	To synthesize evidence about parenting interventions in parents or caregivers of children and adolescents with T1DM, and to evaluate the effect of interventions	CQA	10/11	HQ
Boshoff et al. (40); Australia	Meta-analysis	*n* = 24	P: Parents, mother, father, carer or caregiver of children diagnosed with ASD I: Experience of advocacy Co: Family of children with ASD S: Qualitative research only	DBs: OVID Medline, OVID Nursing, AACM, EMBASE, PsycINFO, ASP, CINAHL, ERIC, HSN/AE, PBSC, Scopus, WoS, CL, IFC IC: In English	To synthesize the experiences of parents advocating for their child with ASD	CQA	10/11	HQ
Delemere and Maguire (28); Ireland	Systematic review	*n* = 16	P: Family/caregivers affected by pediatric cancer I: Connected health technologied interventions O: All outcomes	DBs: PsychInfo, EMBASE, PubMed, WoS IC: In English within the past 10 years	To summarize the efficacy of Connected Health technologies for families/informal caregivers affected by pediatric cancer	CQA	10/11	HQ
Lumsden et al. (41); United Kingdom	Meta-synthesis	*n* = 22	P: Parents of Children with CHD I: Coping (manage an emotional, physical, psychological burden) Co: Family of children with CHD S: An empirical study collecting qualitative data	DBs: MEDLINE, CINAHL, PsycINFO, PubMed, ProQuest, WoS IC: No restriction was placed on language or year	To understand parental coping with their child's CHD	CQA	8/11	MQ
Zhang et al. (42); China	Meta-synthesis	*n* = 8	P: Direct caregivers of children with CP (< 18 years old) I: The real experience and inner needs of caregivers of children with CP Co: Family of children with CP S: Qualitative research	DBs: PubMed, CL, Embase, WoS, SD, CNKI, CBM, VIP, Wanfang IC: In Chinese or English	To systematically review the caregiving experience of family members	CQA	9/11	HQ
Tang et al. (43); China	Meta-analysis	*n* = 11	P: Caregivers of children (< 18 years old) diagnosed with any type of cancer I: PEIs O: Psychosocial and coping outcomes S: RCTs	DBs: Embase, MEDLINE, PsycINFO, CINAHL, Scopus, AMED, JBIEPBD, EBM, BNI, NAHD, ERIC IC: In English	To review the best available evidence to understand the effects of the PEIs on caregivers of children with cancer.	CQA	10/11	HQ
Bourke-Taylor et al. (44); Australia	Meta-analysis	*n* = 17	P: Mothers of children with a disability I: Interventions to improve the mental health O: Wellbeing outcomes S: Quantitative	DBs: OVID Medline, PsychINFO, Embase, Emcare, CCRoCT, CINAHL Plus, Proquest IC: In English	To investigate the effectiveness of interventions that aim to improve the mental health of mothers of children with disabilities.	CQA	10/11	HQ

DBs, databases; IC, inclusion criteria; PICO, population, intervention, comparison/control, outcome; PICoS, population, phenomenon of interest, context, study type; ID, intellectual disability; CQA, Clear quality appraisal; ASD, autism spectrum disorder; WoS, Web of Science; SA, Sociological Abstracts; SSA, Social Services Abstracts; HSN/AE, Health Source Nursing/Academic Edition; AACM, Allied And Complementary Medicine; ASP, Academic Search Premier; PBSC, Psychology and Behavioral Sciences Collection; IHC, Informit Health Collection; SD, Science Direct; JBIEBPD, Joanna Briggs Institute EBP Database; BNI, British Nursing Index; NAHD, Nursing & Allied Health Database; CCRoCT, Cochrane central register of controlled trials; NQA, Non-equality appraisal; T1DM, type 1 diabetes mellitus; RCT, Randomized controlled trial; AAHC, appearance-affecting health conditions; BND, British Nursing Database; CL, Cochrane Library; CHD, Congenital Heart Disease; CP, cerebral palsy; PEI, psychoeducational interventions.

### Critical assessment and risk of bias in included review papers

Details of the critical assessment and risk of bias in review papers were shown in Table 2. Three review papers were evaluated as medium quality, and eleven of them were graded as high quality.

**Table 2:** The critical assessment of included review papers.

**Including review paper (*n* = 14)**	**JBI critical appraisal checklist systematic review**										
	**1**	**2**	**3**	**4**	**5**	**6**	**7**	**8**	**9**	**10**	**11**
Wilson et al. (34)	Y	Y	Y	Y	Y	N	N	Y	N	Y	Y
Gise and Cohen (35)	Y	Y	Y	Y	Y	Y	Y	Y	U	Y	Y
Costa et al. (29)	Y	Y	Y	Y	Y	Y	Y	Y	Y	Y	Y
Kimbell et al. (36)	Y	Y	Y	Y	Y	Y	Y	Y	N	Y	Y
Boehm and Carter (37)	Y	Y	Y	Y	N	Y	N	Y	N	Y	Y
Nuske et al. (38)	Y	Y	Y	Y	Y	Y	Y	Y	N	Y	Y
Rea et al. (39)	Y	Y	Y	Y	N	N	Y	Y	Y	Y	Y
Zhao et al. (30)	Y	Y	Y	Y	Y	Y	Y	Y	N	Y	Y
Boshoff et al. (40)	Y	Y	Y	Y	Y	Y	Y	Y	N	Y	Y
Delemere and Maguire (28)	Y	Y	Y	Y	Y	Y	Y	Y	N	Y	Y
Lumsden et al. (41)	Y	Y	Y	Y	Y	Y	N	Y	N	Y	N
Zhang et al. (42)	Y	Y	Y	Y	Y	Y	Y	Y	N	Y	N
Tang et al. (43)	Y	Y	Y	Y	Y	N	Y	Y	Y	Y	Y
Bourke-Taylor et al. (44)	Y	Y	Y	Y	Y	Y	Y	Y	N	Y	Y

Y, Yes; N, No; U, Unclear; NA, Not applicable. 1. Is the review question clearly and explicitly state?; 2. Were the inclusion criteria appropriate for the review question?; 3. Was the search strategy appropriate?; 4. Were the sources and resources used to search for studies adequate?; 5. Were the criteria for appraising studies appropriate?; 6. Was critical appraisal conducted by two or more reviewers independently?; 7. Were there methods to minimize errors in data extraction?; 8. Were the methods used to combine studies appropriate?; 9. Was the likelihood of publication bias assessed?; 10. Were recommendations for policy and/or practice supported by the reported data?; 11. Were the specific directives for new research appropriate?

### Results of synthesis

Through repeated reading, analysis and interpretation of 14 reviews, we have raised 26 results, and summarized four themes. Finally, evidence to promote social support for caregivers of children with chronic diseases was identified from four key aspects: (i) Intervention content; (ii) Intervention forms; (iii) Intervention time; and (iv) Sources of support. The evidence graded according to the JBI Evidence Pre-Grading System (46) was shown in Supplementary material 4.

#### Intervention content

Within the intervention content, we identified three subthemes: (i) Psychoeducation; (ii) Training or education; and (iii) Attitudes and resources.

#### Psychoeducation

Two meta-analyses explored the effect of psychoeducational interventions on social support (43, 44). According to available evidence, the psychoeducational interventions had no significant effect on social support for caregivers of children with chronic diseases. Specifically, Tang et al. (43) pointed out that the outcome of psychoeducational intervention was not superior to the usual standard of care for social support of caregivers of children with cancer. Standard care mainly referred to routine medical and psychosocial care. Bourke-Taylor et al. (44) also showed no effect of their intervention using psychoeducation approaches on perceived social support for mothers with disabled children.

#### Training or education

One meta-analysis found that Parenting intervention was shown to be beneficial for parents of children with type 1 diabetes mellitus, Specifically, parenting interventions which include supportive parenting training or education programs could help parents of children with type 1 diabetes mellitus ask for positive social support (30).

#### Attitudes and resources

Two systematic reviews summarized the social support condition of caregivers of children with chronic diseases (35, 37). Parents of children with cancer reported that they needed more social support but sought less social support in the process of caring for their children (35). Additionally, there was evidence that parents' perceptions of support availability might be more important than the level of support actually received. In other words, whether parents felt they had a supportive relationship might be more important than the actual amount of support they received (37).

#### Intervention forms

Within the intervention forms, we identified three subthemes: (i) Supportive groups; (ii) online; and (iii) Community organizations/teams.

##### Supportive groups

Two systematic reviews and one meta-analysis synthesized the impact of the group-based interventions on social support for caregivers of children with chronic diseases (34, 39, 44). No consensus had been reached. Bourke-Taylor et al. (44) indicated that group support therapy which relied on peer engagement and group interactions with discussions around support, coping and information sharing had not been shown to increase the perceived level of social support due to insufficient research. Wilson et al. (34) reported the effect of group-based interventions aimed at strengthening social relationships was inconclusive. Only one review of parent-involved therapeutic concentration camps found that parents report camp was a place for providing social support for families of children with chronic health conditions (39).

##### Online

A systematic review synthesized the role of Connected Health technologies in supporting families affected by pediatric cancer (28). Internet-based health technologies could influence the psychosocial needs of caregivers, provided them with psychosocial support, and reduced the adverse effects of social isolation (28). Another systematic review also identified that support could be provided through networks (38).

##### Community organizations/teams

A systematic review synthesized coping strategies for caregivers of children with autism spectrum disorder during the new school transitions and concluded that community-based organizations and supportive teams could provide support (38).

#### Intervention time

A systematic review synthesized early family intervention programs in support of parents with cleft lip and palate (29). Early intervention programs aiming to support parents in adapting to having a child with a disability were implemented when the child was 6, 12, and 18 months, which demonstrated moderate evidence for the effectiveness of increasing perceived spousal emotional support (29).

#### Sources of support

Within the sources of support, we identified four subthemes: (i) Family members; (ii) Informal people outside the home; (iii) Professionals; and (iv) Faith/spirituality.

##### Family members

Three Meta-analyses and one systematic review affirmed the role of family members in providing social support (35, 40–42). Family and significant others were the most prevalent sources of support (35). Caregivers expected support from family and society (42). Parents of children with autism spectrum disorder described a strong network of support to enable advocacy, such as partners and extended family (40). For many parents whose children with congenital heart disease, close families, particularly their children's grandparents, became an invaluable source of support to help parents cope with hard times (41). What's more, parents could get emotional and practical support from someone close to them, especially couples (41).

##### Informal people outside the home

Two Meta- analyses and two systematic reviews integrated informal staff support for caregivers of children with chronic diseases (36, 37, 40, 41). A lot of support, including information support, could be found in informal relationships and emotional support from informal relationships outside the family system was a particularly important resource for parents (37). These informal sources of support mainly include peers, friends and others in the social or school system. While most parents visited some forms of support, they still admitted that others didn't really understand what they were going through unless they had gone through a similar experience themselves, so, parents emphasized that connecting with peers who had the same situation constituted an important source of emotional and practical support (36). Many problems could be solved, and their heads kept clear with the help of peers (41). Friends could be as validators, sounding boards and observers with constructive support (40). Social and school system support is available in rural communities (40).

##### Professionals

A meta-synthesis affirmed the role of professionals among parents of children with congenital heart disease (41). Parents reported that the honesty, reassurance and information which professionals provided helped them to understand their children's condition more, and in turn cope better with what they faced as a family (41).

##### Faith/spirituality

Spiritual support comes mainly from people of faith. Parents turned to faith, religion and often prayer to call upon a “higher power” for support, and felt comforted when procedures were successful, attributing this to divine intervention (41).

## Discussion

Based on the review and thematic synthesis of the included review papers (*n* = 14), we have identified four key aspects of improving social support for caregivers of children with chronic diseases: intervention content, intervention forms, intervention time, and the sources of support.

### Intervention content

Summarizing current evidence, we divided the intervention content of social support for caregivers of children with chronic diseases into three main aspects: psychoeducation, training or education, and attitudes and resources. Despite this, the intervention content of improving social support is still relatively limited. The Psychoeducational intervention is a non-pharmacological approach that involves information giving and receiving, concerns about emotions, psychological needs and family relationships (47, 48). Bourke-Taylor et al. (44) and Tang et al. (43) pointed out that psychoeducational interventions cannot play a role in improving the social support of caregivers of children with cancer or disability. In a study of caregivers of patients with lung cancer, psychological education interventions also showed no effect on the level of social support for caregivers (49). In other research, psychoeducational interventions can increase caregivers' knowledge of diseases, strengthen their stress coping skills, improve psychological outcomes and make better quality of life (49, 50). As you can see, the effectiveness of psychoeducational interventions have been demonstrated in many of the above areas. However, the role of psychoeducational interventions on social support remains to be further explored, and extensive original research is still needed. Training or education could help increase the level of social support (30). Specifically, it focuses on helping parents of children with chronic diseases feel positively supported and providing them with practical parenting guidance, available information and resources. It is worth noting that, caregivers represent they rarely actively seek social support, although they acknowledge that having available social connections is more important than actual support (35, 37). To a certain extent, this means that caregivers of children with chronic diseases have not established a positive attitude to deal with the current difficulties, while only limited to passively accepting existing assistance. This is even more dangerous in areas with inadequate medical resources, because there are not enough personnel to identify families in need. If the family does not actively seek help and does not actively use the existing social resources, the family will encounter more difficulties. They will also feel lonelier and more isolated. Therefore, it is necessary to add how to actively seek social support in the content of future interventions to guide caregivers to use intentional relationships and actively seek available resources to get social support.

### Intervention forms

Intervention forms of social support for caregivers of children with chronic diseases were integrated into three types: group, online and community organizations/teams. Support groups refer to approaches that relied on peer engagement and group interactions with discussions around supports, coping and information sharing (34, 39, 44). Although existing evidence is not yet consistent that group intervention has a positive impact on social support for caregivers of children with chronic disease (34, 39, 44). However, previous research showed support groups can expand and strengthen social support networks for disabled elderly caregivers (51). The form of group intervention can greatly unite caregivers of children with chronic diseases and promote their participation and interaction. Many studies have confirmed that group support therapy can help caregivers to solve psychological burdens and adapt to the disease experience, and at the same time, caregivers have improved social functions, physical pain, general health, emotional health, role limitations caused by emotional problems (52, 53). In summary, we have reason to think that the group intervention method is still worthy of promotion in clinical practice. The online form refers to ways that rely on the Internet, through smartphone apps, online web pages, telemedicine and online groups (28). The biggest advantage of this form is that it is not affected by distance, allowing a wider range of families to participate. The online intervention focuses on providing information on disease management resulting in a significant reduction in parental anxiety and uncertainty and an increase in social functioning and knowledge (21, 54). Compared with the group intervention form, the online form is more convenient and faster. Caregivers of children with chronic diseases can obtain more comprehensive information resources according to their needs, but the real-time interaction and practice are weaker than the group form. It cannot be ignored that most interventions for caregivers of children with chronic diseases usually take 3 to 6 months (55). With such a long-term intervention, it is a challenge for many caregivers to ensure that they can participate on time. In this situation, considering the large daily burden of caregivers of children with chronic diseases, the energy and time spent on taking care of their children, and the actual effect that they want to achieve, it will be a good choice in the future to integrate these two forms. For example, in the first stage, online system learning and guidance are carried out with the help of connected health technologies. Meanwhile, problems and difficulties encountered by caregivers of children with chronic diseases are collected. The second stage is to carry out offline practice in groups, organize the learning and application of various parenting skills in the form of entertainment activities, and gather caregivers in similar situations. They can exchange experiences and provide each other with the required resources. Parenting issues or psychological confusions faced by caregivers can be guided face-to-face. This hybrid approach may be a choice for more caregivers of children with chronic diseases in the future, and it is also a good form for social workers and healthcare professionals to carry out interventions.

### Intervention time

Early family intervention improves perceived spousal emotional support for caregivers of children with chronic diseases. The “Early” is the stage when caregivers of children with chronic diseases frequently experience various problems, such as psychological maladaptation, frequent anxiety and depression. Interventions for caregivers at an early stage also have positive implications for promoting adaptation, reducing threats, increasing confidence, and reducing emotional distress, anxiety, and depression (29). Early help is more like timely rain. Therefore, we advocate early intervention for caregivers of children with chronic diseases to promote the perception of spouse support, and to help them establish an orderly parenting state, a stable and positive attitude, and positive coping skills.

### Sources of support

The results of our umbrella review also suggest that caregivers of children with chronic diseases can receive support from family members, friends, peers, community, self-belief, and professionals (35–38, 40–42), which is approximately consistent with the composition of the perceived social support scale. In general, informal people are an important source of social support for caregivers of children with chronic illness, especially family members and peers. The family is the living unit of the caregivers of children with chronic diseases. A close family relationship is a favorable way to relieve negative emotions and provide confidence and support. When caregivers of children with chronic diseases are faced with stressful events, support from family members can greatly relieve the pressure of parenting and economics (56–58). As special family members, partners have more contact with caregivers, and their positive emotional feedback and support are favorable factors for caregivers to cope positively (59, 60). Therefore, we suggest that partners of caregivers of children with chronic diseases should actively participate in parenting, maintain a stable mood, encourage and communicate with each other, and establish a close relationship of dependence. In addition to family members, available informal relationships outside of the home system are also a good source of support. Peers are one important part of it. It's worth mentioning that while most parents caring for children with chronic diseases access some sources of support, they still admit that others don't really understand what they're going through unless they've gone through a similar experience themselves (41). This suggests that peer support is more meaningful for caregivers. These people with similar experiences of diseases or psychosomatic conditions help each other in social and emotional aspects, which can give each other more encouragement and confidence to overcome difficulties together (61, 62). Support provided by these informal groups tends to be emotional, and it is more about improving the mentality of caregivers, and providing spiritual dependence and confidence. We recommend providing psychological counseling for family members, especially couples. When patients receive treatment, medical institutions can provide them with communication platforms among peers.

### Limitations

Some limitations of this umbrella review need to be considered. Firstly, this review did not utilize all available databases (e.g., CINAHL, MEDLINE). Therefore, some evidence may have been ignored. Secondly, only articles published in English and Chinese were included, which might have led to selection bias as articles published in different languages were not considered. Thirdly, although we followed published guidelines for systematic reviews (31), we did not register our search protocol prior to the start of the review. Fourthly, quality assessments were performed using the Joanna Briggs Institute, a useful tool, although its reliability could be improved by additional assessment of the methodological quality of included studies (63). Finally, there is still a lack of research on psychoeducational interventions and group interventions for caregivers of children with chronic diseases, and their role in promoting the level of social support for caregivers of children with chronic diseases is not clear enough. At the same time, due to the strong heterogeneity of the included studies, there is not enough quantitative data for quantitative integration.

### Implications for practice

The findings of the umbrella review showed that four main aspects can be taken to optimize social support for caregivers of children with chronic diseases in clinical practice. Healthcare professionals and social workers should enhance their expertise to provide available resources and health guidance for caregivers. It is also extremely important to help caregivers of children with chronic diseases identify available support relationships, especially family members and peers. This means that it is meaningful to establish a good family relationship and create a harmonious family atmosphere, and it is necessary to implement psychological counseling between the couple to establish a good supportive relationship. We encourage medical institutions and interveners to intervene early in families of children with chronic diseases, integrate diverse and effective intervention content, and use different forms at different time periods to help the caregivers of children with chronic diseases for the greatest benefit.

## Conclusion

Social support plays an important role in improving mental health of caregivers of children with chronic diseases and in promoting active parenting. The findings of this umbrella review suggest that a combination of effective and diverse intervention content and forms to improve social support for caregivers of children with chronic diseases is recommended. In general, it is pivotal to follow the four aspects on how to improve social support for caregivers of children with chronic diseases, which include the content, forms, timing and the sources of social support. Specifically, the use of a combination of differing interventions, especially for early family, including content of parenting training or education, attitude building and resource provision, which can implement online, are most effective aiming at improving social support for caregivers of children with chronic diseases. Nonetheless, evidence for increasing the level of social support is still limited, and original interventional research and quantitative evidence integration for caregivers of children with chronic diseases is still needed.

## Data availability statement

The original contributions presented in the study are included in the article/Supplementary material, further inquiries can be directed to the corresponding author.

## Author contributions

JY: conceptualization, formal analysis, methodology, investigation, writing—original draft, and writing—review and editing. LL: conceptualization, methodology, and writing—original draft. YG: supervision, resources, and writing—review and editing. WW: methodology, resources, investigation, and writing—review and editing. LY: conceptualization, methodology, visualization, investigation, formal analysis, funding acquisition, project administration, and writing—review and editing. All authors contributed to the article and approved the submitted version.

## Funding

This study was supported by the project of Education Department of Liaoning Province (LJKR0281).

## Conflict of interest

The authors declare that the research was conducted in the absence of any commercial or financial relationships that could be construed as a potential conflict of interest.

## Publisher's note

All claims expressed in this article are solely those of the authors and do not necessarily represent those of their affiliated organizations, or those of the publisher, the editors and the reviewers. Any product that may be evaluated in this article, or claim that may be made by its manufacturer, is not guaranteed or endorsed by the publisher.
