# Supplementary material for: Interventions and strategies to improve social support for caregivers of children with chronic diseases: An umbrella review
Source: Front Psychiatry. 2022 Sep 23;13:973012. doi: 10.3389/fpsyt.2022.973012 (PMC9537372; doi:10.3389/fpsyt.2022.973012)
Supplement: Supplementary file 1 [file Data_Sheet_1.docx]

# Supplementary material 1: Written protocol

# Review question

What interventions and strategies can improve social support among caregivers of children with chronic diseases?

# Searches

- PubMed
- Embase
- Web of Science
- OVID
- CNKI
- CBM
- Wanfang
- Cochrance library
- Timeframe: until November 2021
- Language: English and/or Chinese

# Types of study to be included

Systematic review, meta-analysis

# Condition or domain being studied

Health psychology

# Participants/ population

Participants to be included are those who are taking care of children with chronic diseases.

Who are taking care of children can be described as caregivers, parents, fathers and /or mothers.

Children with chronic diseases are defined as (Mokkink et al., 2008):

- It occurs in children aged 0 up to 18 years;
- The diagnosis is based on medical scientific knowledge and can be established using reproducible and valid methods or instruments according to professional standards;
- It is not (yet) curable or, for mental health conditions, if it is highly resistant to treatment;
- It has been present for longer than three months or if it will, very probably, last longer than three months;
- or if it has occurred three times or more during the past year and will probably recur again.

Due to the wide variety of chronic diseases in children, including: diabetes, congenital deformities, asthma, cancer, kidney disease, pervasive developmental disorders, etc. If the disease is specifically defined, it will affect the recall rate. Therefore, in the database search, no specific disease was defined. This will be taken into account during article screening.

# Intervention/Phenomena of interest

interventions or strategies aimed at social support. Specifically, interventions or strategies for social support of caregivers of children with chronic diseases could be included in the review.

During the search, in order to ensure the recall rate of the literature, no restrictions were placed on the intervention or strategy. We will focus on this during the literature screening phase.

# Comparator(s)/ control

Not applicable

# Context

In the families of children with chronic diseases under the age of 18

# Outcome(s)

Social support

# Data selection

Author [5] identified search terms from previous literature, and Author [1] consulted to refine search terms. After Author [1] and Author [5] defined final search terms, Author [5] developed search strings carried out searches in relevant databases.

Author [1] and Author [2] will screen titles and abstracts for inclusion in Endnote X7.7, an online screening and study extraction tool, and will include articles that have, or might have, social support as the dependent variable in samples in which participants are taking care of children with chronic diseases. Discrepancies will be discussed and resolved by the authors in conflict. Author [1] and Author [5] will then screen full-text articles for inclusion, and discrepancies will be discussed and resolved by discussion between these two authors.

# Data extraction and coding

Author [1] and Author [4] took charge of data extraction on basis of the predeﬁned criteria and was checked by other authors. Data extracted will include Basic information of the article , demographic information, types of childhood chronic diseases, study design/methodology and the specific topic and purpose of the article. When necessary, we will contact study authors to obtain information.

Data to be extracted is as follows:

- - Authors, year, and country
  - Study type (systematic review, meta-analysis),
  - How many articles were included
  - Search strategy
  - PICO(S)/PICo
  - Age of children with chronic diseases
  - Specific chronic disease types
  - Person who takes care
  - Methodological quality assessment

The synthesis of the data was implemented as that: Firstly, the ﬁrst author sought for free codes in the articles involved line by line. Secondly, primary subthemes were raised by integrating these free codes. Thirdly, secondary themes were developed by comparing and analyzing the primary subthemes. Last, all the co-authors discussed and reached the consensus for the above outcomes.

# Risk of bias (quality) assessment

Quality assessments will be carried out using the Joanna Briggs Institute Critical Appraisal Checklist for Systematic Reviews and Research Syntheses (Aromataris et al., 2015) for assessing risk of bias, and study quality will be reported in presentations at the manuscript that we will write as part of dissemination of knowledge.

# Strategy for data synthesis

Findings are reported using narrative synthesis.

# Analysis of subgroups or subsets

Not applicable

# Dissemination plans

We plan to present this work through publishing a manuscript with the findings.

# Contact details for further information

The Corresponding Author:

Author [5]

See Tittle page

# Organisational affiliation of the review

See Tittle page

# Review team

Author [1]

Author [2]

Author [3]

Author [4]

Author [5]

See Details in the Tittle page

# Anticipated or actual start date

November 15, 2021

# Anticipated completion date

May 24, 2022

# Funding sources/sponsors

This work was supported by the project of Education Department of Liaoning Province (LJKR0281)

# Conflicts of interest

Have no conflict of interest to disclose

# Language

English

# Country

China

# Subject index terms

Social support; caregiver; parents; father; mother; systematic review; Meta analysis;

# Stage of review

Completed

# Date of registration in PROSPERO

Not registered

# Date of publication of this revision

Not registered

| Stage of review at time of this submission | **Started** | **Completed** |
| --- | --- | --- |
| Preliminary searches | Yes | Yes |
| Piloting of the study selection process | Yes | Yes |
| Formal screening of search results against eligibility criteria | Yes | No |
| Data extraction | No | No |
| Risk of bias (quality) assessment | No | No |
| Data analysis | No | No |

| **Supplementary material 2:** Search Terms | | |
| --- | --- | --- |
| Datebase | Search strategy | Records |
| Pubmed | ("parents"[MeSH Terms] OR "caregivers"[MeSH Terms]) AND "social support"[MeSH Terms] AND ("Meta-Analysis"[Publication Type] OR "systematic review"[Publication Type] | 357 |
| Web of science | (TI=(Parent*) OR TI=(Caregiver*)) AND(TS=(social support) AND (TS=(Meta-Analysis) OR TS=(systematic review) OR TI=(Meta))) | 573 |
| Embase | ('parent'/exp OR 'biological parent' OR 'parent' OR 'parents') AND ('social support'/exp OR 'social support' OR 'support, social') AND ('meta analysis'/exp OR 'systematic review'/exp) | 281 |
| OVID | (parent*.at. or parent*.mh. or caregiver*.mh. or caregiver*.at.) AND (social support*.at. or social support*.mh. or social support*.ab.) AND ((Meta or systematic review).at. OR (Meta or systematic review).mh.) | 475 |
| CNKI | (SU %= '社会支持' OR KY = '社会支持' OR TI = '社会支持') AND (SU %= '父母'+'父亲'+'母亲'+'照顾者'+'家庭' OR KY = '父母'+'父亲'+'母亲'+'照顾者'+'家庭' OR TI = '父母'+'父亲'+'母亲'+'照顾者'+'家庭') AND (SU %= 'Meta'+'系统综述'OR KY = 'Meta'+'系统综述'OR TI = 'Meta'+'系统综述') | 54 |
| Wanfang | (主题:(父母 or 父亲 or 母亲 or 照顾者 or 家庭) or 题名或关键词:(父母 or 父亲 or 母亲 or 照顾者 or 家庭)) and (主题:(社会支持) or 题名或关键词:(社会支持) ) and (题名或关键词:((Meta) or 系统综述)) | 116 |
| CBM | "社会支持"[核心字段:智能] AND  ("Meta"[常用字段:智能] OR "系统综述"[常用字段:智能]) | 44 |
| *Note.* TI = title; TS =topic; SU = subject; KY = key words. | | |

| **Supplementary material 3:** Exclusion Criteria For Full-Text Articles | | |
| --- | --- | --- |
| Author, Year of Publication | Title | Reason for exclution |
| Widman et al., 2020 | Supports for Postsecondary Students with Autism Spectrum Disorder: A Systematic Review | The study population is Postsecondary Students with Autism Spectrum |
| Olive et al., 2017 | The Effect of Internet Group Support for Caregivers on Social Support, Self-Efficacy, and Caregiver Burden: A Meta-Analysis | The study population is adult family caregivers of adult patients |
| McKechnie et al., 2014 | Effectiveness of computer-mediated interventions for informal carers of people with dementia-a systematic review | The study population is not caregivers of children with chronic diseases |
| Mason et al., 2008 | Telephone interventions for family caregivers of patients with dementia: what are best nursing practices? | The study population is not caregivers of children with chronic diseases |
| Lopez-Hartmann et al., 2012 | The effect of caregiver support interventions for informal caregivers of community-dwelling frail elderly: a systematic review | The study population is caregivers of community-dwelling frail elderly |
| Lins et al., 2014 | Efficacy and experiences of telephone counselling for informal carers of people with dementia | The study population is not caregivers of children with chronic diseases |
| Kaltenbaugh et al., 2015 | Using Web-Based Interventions to Support Caregivers of Patients With Cancer: A Systematic Review | The study population is caregivers of adult patients with cancer aged 18  years or older |
| Jeon et al., 2019 | Systematic Review on Intervention Program for Family Caregivers of People with Dementia | The study population is not caregivers of children with chronic diseases |
| Gao et al., 2021 | Supporting caregivers of people with dementia: A systematic review of guidelines | The study population is not caregivers of children with chronic diseases |
| Dam et al., 2016 | A systematic review of social support interventions for caregivers of people with dementia: Are they doing what they promise? | The study population is not caregivers of children with chronic diseases |
| Clayton et al., 2019 | Enhancing Social Support Among People with Cardiovascular Disease: a Systematic Scoping Review | The study population is not caregivers of children with chronic diseases |
| Cheng et al., 2020 | A comprehensive meta-review of systematic reviews and meta-analyses on nonpharmacological interventions for informal dementia caregivers | The study population is not caregivers of children with chronic diseases |
| Chen et al., 2016 | Non-pharmacological interventions for caregivers of patients with schizophrenia: A meta-analysis | The study population is caregivers of patients with  schizophrenia |
| Brodat et al., 2003 | Meta-analysis of psychosocial interventions for caregivers of people with dementia | The study population is not caregivers of children with chronic diseases |
| Bademli et al., 2011 | Family to family support programs for the caregivers of schizophrenia patients: a systematic review | The study population is caregivers of patients with  schizophrenia |
| Aldehaim et al., 2016 | The Impact of Technology-Based Interventions on Informal Caregivers of Stroke Survivors: A Systematic Review | The study population is informal caregivers of Stroke Survivors |

| **Supplementary material 3:** Exclusion Criteria For Full-Text Articles (Continued) | | |
| --- | --- | --- |
| Author, Year of Publication | Title | Reason for exclution |
| Abrahams et al., 2018 | Effectiveness of interventions for co-residing family caregivers of people with dementia: Systematic review and meta-analysis | The study population is not caregivers of children with chronic diseases |
| Nieuwboer et al., 2013 | Online programs as tools to improve parenting: A meta-analytic review | The study population is parents of children aged between -9 months (pregnancy) and 21 years |
| Niela-Vilen et al., 2014 | Internet-based peer support for parents: A systematic integrative review | The study population is mothers and/or fathers or pregnant women |
| Caton et al., 2019 | Internet use for family carers of people with intellectual disabilities: A literature review and thematic synthesis | The study population is carers of people with intellectual disabilities. |
| Applebaum et al., 2013 | Care for the cancer caregiver: A systematic review | The study population is caregivers of people with illness/disease |
| Melead et al., 2020 | Positive contributions among parents of children on the autism spectrum: A Systematic review | The outcome variables were positive contributions of caregivers |
| Patterson et al., 2012 | A systematic review of training programs for parents of children with autism spectrum disorders: Single subject contributions | The outcome variables are communication and social development in children with autism spectrum disorders |
| Babic et al., 2013 | Early intervention experiences of parents of children with developmental disabilities | The language is inconsistent |
| Dawson-Squibb et al., 2019 | Scoping the evidence for EarlyBird and EarlyBird Plus, two United Kingdom-developed parent education training programmes for autism spectrum disorder | Methodology: A scoping review |
| de Verdie et al., 2020 | Blindness and Autism: Parents’ Perspectives on Diagnostic Challenges, Support Needs and Support Provision | Methodology: A qualitative interview-based design |
| Bray et al., 2017 | Parent-to-parent peer support for parents of children with a disability: A mixed method study | Methodology: A mixed method study design (qualitative and quantitative) |
| Newman et al., 2019 | The role of internet-based digital tools in reducing social isolation and addressing support needs among informal caregivers: a scoping review | Methodology: A scoping review |
| Lemacks et al., 2013 | Insights from parents about caring for a child with birth defects | Methodology: Question guidance and advice |
| Hedov et al., 2010 | Better support to first-time parents of children with life-long functional disabilities. Proposal to new guidelines | Methodology: Proposal to new guidelines |
| Pate et al., 2015 | Support group for parents coping with children with type 1 diabetes | Methodology: A quantitative study |
| Wei et al., 2012 | Support groups for caregivers of intellectually disabled family members: effects on physical-psychological health and social support | Methodology: An experimental, preintervention postintervention control group study design |
| Chien et al., 2009 | The effectiveness and active ingredients of mutual support groups for family caregivers of people with psychotic disorders: A literature review | Methodology: A literature review |
| Ugunasingha et al., 2019 | Interventions to improve outcomes for caregivers of children with food allergies: a systematic review | The type of disease is incompatible |

| **Supplementary material 4:** Pre-Grading of Evidences | | | | | |
| --- | --- | --- | --- | --- | --- |
| Oder | Author | Category | Content | Evidence details | Grading |
| 1 | Tang et al., 2020; Bourke-Taylor et al., 2021; Zhao et al., 2019; Gise et al., 2021; Boehm et al., 2016 | Intervention contents | Psychoeducation  Training or education  Attitudes and resources | • The outcomes from psychoeducational interventions were not superior to the usual standard of care for social support.  • No effect of their intervention using psychoeducation approaches on perceived social support.  • Parenting interventions could help parents ask for positive social support.  • Parents generally reported more perceived social support, but less social support seeking.  • Often the availability of social connections is more important than the actual amount of support received | Tang et al., 2020, 1a    Bourke-Taylor et al., 2021, 1b  Zhao et al., 2019, 1a  Gise et al., 2021, 4a  Boehm et al., 2016, 4a |
| 2 | Bourke-Taylor et al., 2021; Wilson et al., 2014; Rea et al., 2019; Delemere et al., 2021; Nuske HJ | Intervention forms | Supportive groups  Online | • Group support therapy ( relied on peer engagement and group interactions with discussions around supports, coping and information sharing) has not been shown to increase perceived levels of social support due to insufficient research.  • Group-based interventions aimed at strengthening social relationships was inconclusive.  • Parents report camp to be a place for providing social support.  • Connected Health technologies are beneficial to satisfy psychosocial needs and reduce social isolation.  • Connected Health technologies are beneficial for providing psychosocial support. | Bourke-Taylor et al., 2021, 1c  Wilson et al., 2014, 2a  Rea et al., 2019, 2a  Delemere et al., 2021, 1c  Delemere et al., 2021, 1a |

| **Supplementary material 4:** Pre-Grading of Evidences (Continued) | | | | | |
| --- | --- | --- | --- | --- | --- |
| Oder | Author | Category | Content | Evidence details | Grading |
| 2 | Bourke-Taylor et al., 2021; Wilson et al., 2014; Rea et al., 2019; Delemere et al., 2021; Nuske et al., 2019 | Intervention forms | Community organizations/teams or networks | • Community organisations, support teams or networks can provide support | Nuske et al., 2019, 1 |
| 3 | Costa et al., 2021 | Intervention time | Early Family Intervention Program | • Early Family Intervention Program can increase perceived spousal emotional support | Costa et al., 2021,1b |
| 4 | Boshoff et al., 2016; Lumsden et al., 2019; Zhang et al., 2020; Gise et al., 2021; Boehm et al., 2016; Kimbell et al., 2021 | Sources of support | Family members  Informal people outside the home | • Parents described a strong network of support to enable advocacy, such as partners and extended family.  • For many parents, close family, particularly their child’s grandparents, became an invaluable source of support to help parents cope.  • Caregivers expect family and social support  • Family and significant others are the most prevalent sources of support.  • Parents reported turning to someone close to them (within the Couple) for emotional and practical support.  • Emotional support through informal relationships outside the family system is a particularly important resource for parents.  • A lot of support, including information support, can be found in informal relationships. | Boshoff et al., 2016, 1  Lumsden et al., 2019, 1  Zhang et al., 2020, 2  Gise et al., 2021, 4a  Lumsden et al., 2019, 1  Boehm et al., 2016, 4a    Boehm et al., 2016, 4a |

| **Supplementary material 4:** Pre-Grading of Evidences (Continued) | | | | | |
| --- | --- | --- | --- | --- | --- |
| Oder | Author | Category | Content | Evidence details | Grading |
| 4 | Boshoff et al., 2016; Lumsden et al., 2019; Zhang et al., 2020; Gise et al., 2021; Boehm et al., 2016; Kimbell et al., 2021 | Sources of support | Informal people outside the home  Professionals  Faith/spirituality | • connecting with other parents who had a child with type 1 diabetes constituted an important source of emotional and practical support.  • While most parents visit some form of support, they still admit that others don't really understand what they're going through unless they've gone through a similar experience themselves  • Many parents use support forums to get support from peers in similar experiences to solve many problems and keep their heads clear.  • friends are as validators, sounding boards and observers with constructive support  •Social and school system support is available in rural communities  • Parents reported that the honesty, reassurance and information that professionals provided helped them to understand their child’s condition more, and in turn cope better with what they faced as a family.  • Parents turned to faith, religion and often prayer to call upon a ‘higher power’ for support, and felt comforted when procedures were successful, attributing this to divine intervention. | Kimbell et al., 2021, 1  Lumsden et al., 2019, 1  Lumsden et al., 2019, 1    Boshoff et al., 2016, 1  Boshoff et al., 2016, 1  Lumsden et al., 2019, 1  Lumsden et al., 2019, 1 |
